# Myeloperoxidase and eosinophil cationic protein in serum and sputum during antibiotic treatment in cystic fibrosis patients with Pseudomonas aeruginosa infection

**DOI:** 10.1155/S0962935195000457

**Published:** 1995-07

**Authors:** B. Niggemann, T. Stiller, K. Magdorf, U. Wahn

**Affiliations:** 1 University Children's Hospital (KAVH) Virchov-Klinikum of Humboldt University Berlin Heubnerweg D-14059 Germany

## Abstract

I order to study the time-course of myeloperoxidase (MPO) and eosinophil cationic protein (ECP) as parameters for monitoring inflammation in cystic fibrosis (CF), we investigated ten patients during both a 14-day intravenous antibiotic treatment and a corresponding self control. Modified Shwachman-Kulczycki score improved significantly (p < 0.008), C-reactive protein (CRP) levels decreased significantly (p < 0.05) during antibiotic treatment, while in the control phase there were no significant differences. Lung function parameters did not change significantly during antibiotic treatment or control phase. Serum MPO concentration (p < 0.006) and peripheral blood neutrophil granulocyte counts (p < 0.04) decreased significantly during antibiotic treatment, but not during the control phase. Sentm ECP concentration showed a tendency to decrease during antibiotic treatment, but this failed to reach significance. In general, sputum concentrations of MPO and ECP Were 500- to 1000-fold higher than in serum. However, neither MPO nor ECP in sputum showed a significan variation over time during antibiotic treatment or control phase. From our data we conclude that: (1) measurements of MPO, neutrophils and CRP in peripheral blood do correlate with clinical parameters such as the modified Shwachman-Kulczycki score; (2) neutrophils and MPO seem to reflect inflammatory changes induced by antibiotic treatment; (3) eosinophils may play a role in CF by an enhanced ‘releasability’ and (4) Sputum measurements of mediators of inflammation cannot be recommended.


Mediators of Inflammation 4, 282–288 (1995)

### Myeloperoxidase and eosinophil cationic protein in serum and sputum during antibiotic treatment in cystic fibrosis patients with *Pseudomonas aeruginosa* infection

B. Niggemann,[CA] T. Stiller, K. Magdorf and U. Wahn

University Children's Hospital (KAVH), Virchov-Klinikum of Humboldt University, Heubnerweg 6, D-14059, Berlin, Germany

[CA]Corresponding Author

**In order to study the time-course of myeloperoxidase (MPO) and eosinophil cationic protein (ECP) as parameters for monitoring inflammation in cystic fibrosis (CF), we investigated ten patients during both a 14-day intravenous antibiotic treatment and a corresponding self control. Modified Shwachman–Kulczycki score improved significantly ($p < 0.008$), C-reactive protein (CRP) levels decreased significantly ($p < 0.05$) during antibiotic treatment, while in the control phase there were no significant differences. Lung function parameters did not change significantly during antibiotic treatment or control phase. Serum MPO concentration ($p < 0.006$) and peripheral blood neutrophil granulocyte counts ($p < 0.04$) decreased significantly during antibiotic treatment, but not during the control phase. Serum ECP concentration showed a tendency to decrease during antibiotic treatment, but this failed to reach significance. In general, sputum concentrations of MPO and ECP were 500- to 1000-fold higher than in serum. However, neither MPO nor ECP in sputum showed a significant variation over time during antibiotic treatment or control phase. From our data we conclude that: (1) measurements of MPO, neutrophils and CRP in peripheral blood do correlate with clinical parameters such as the modified Shwachman–Kulczycki score; (2) neutrophils and MPO seem to reflect inflammatory changes induced by antibiotic treatment; (3) eosinophils may play a role in CF by an enhanced 'releasability' and (4) Sputum measurements of mediators of inflammation cannot be recommended.**

**Key words:** Cystic fibrosis, Eosinophil cationic protein, Myeloperoxidase, *Pseudomonas aerugonisa*

### Introduction

Patients with cystic fibrosis (CF) suffer from chronic *Pseudomonas aeruginosa* infection and a dramatic neutrophil recruitment into the lungs. This results in a proteinase imbalance and a subsequent progressive proteolytic destruction of lung connective tissue.[1–3] Deterioration of lung function and parameters of inflammation (e.g., C-reactive protein CRP) parallels the time-course of progressive host inflammatory responses to chronic *Pseudomonas aeruginosa* infection.[4]

Myeloperoxidase (MPO), as a neutrophil granulocyte-specific inflammatory mediator, could be shown to have cytotoxic properties to the lung epithelium *in vitro*.[5] Other neutrophil granule products correlate with lung function parameters and CRP in acute exacerbations in CF.[6]

Eosinophils and their granule proteins play a major role for chronic inflammation in bronchial asthma, but there is controversy about the role of the eosinophils in CF. While an Austrian working group saw evidence for an eosinophilic activation in CF,[7] other authors did not find elevated numbers of eosinophils in post-mortem CF lungs.[8]

Because there is little information concerning the role of the inflammatory mediators MPO and eosinophil cationic protein (ECP) as parameters for monitoring inflammation in CF patients, we studied these mediators in serum and sputum during a 14-day intravenous antibiotic treatment and a 14-day in-patient control phase and compared them with clinical parameters.

### Patients and Methods

*Patients:* Ten patients (three males, seven females) aged 14 to 44 years (mean age 24 years) with cystic fibrosis were enrolled in the study. Patient data are shown in Table 1. Diagnosis of

© 1995 Rapid Science Publishers

Table 1. Patient data at entry into study

| Patient no. | Sex | Age (years) | Weight (kg) | Height (cm) | C–N score | Total IgE (kU/l) | Atopic |
|---|---|---|---|---|---|---|---|
| 1 | M | 14 | 28.9 | 149 | 23 | 278 | no |
| 2 | F | 16 | 37.8 | 153 | 20 | 132 | no |
| 3 | M | 29 | 65.7 | 178 | 22 | 402 | yes |
| 4 | F | 25 | 49.8 | 158 | 16 | 1457 | yes |
| 5 | F | 24 | 38.8 | 153 | 15 | 305 | yes |
| 6 | F | 26 | 62.0 | 174 | 14 | 103 | no |
| 7 | F | 44 | 44.2 | 145 | 14 | 5 | no |
| 8 | M | 19 | 45.6 | 168 | 10 | 792 | yes |
| 9 | F | 20 | 44.0 | 161 | 14 | 172 | no |
| 10 | F | 28 | 41.0 | 160 | 23 | 10 | no |
| mean | | 24 | 43.8 | 160 | 17 | 366 | |

C–N score = Chrispin–Norman score; atopic = specific IgE against inhalative allergens.

CF was established by typical clinical manifestations of the disease and confirmed by positive sweat tests (sweat sodium concentration above 70 mmol/l). In all patients, chronic *Pseudomonas aeruginosa* infection was demonstrated by repetitive positive sputum cultures. Four of the ten patients (nos. 3, 4, 5 and 8) were atopic, on the basis of proof of specific IgE against common inhalative allergens. Total IgE of all patients ranged from 5 kU/l to 1457 kU/l (mean 363.2 kU/l). Informed written consent was given by all patients or their parents. Antibiotic treatment was chosen according to their antibiotic resistance pattern: seven patients received a combination of ceftazidime and tobramycin; two patients received imipenem and tobramycin; and one patient received ticarcillin and clavulanic acid.

*Study design:* Patients were admitted to hospital in a cross-over design both for 14 days of intravenous antibiotic treatment against *Pseudomonas aeruginosa*[9] and (4 to 6 weeks before or after) for the same period without antibiotic treatment. Therefore, each patient served as their own control. Time of admittance was chosen when patients were free of acute exacerbations. During both hospitalizations, usual conservative treatment, such as intensive physiotherapy, inhalation therapy etc., was performed identically. Blood and sputum samples were collected in the morning of days 1, 3, 5, 8, 10, 12 and 15 between 08:00 and 09:00. Lung function tests were obtained on the same days.

*Methods:* The clinical severity of CF was assessed by the Shwachman–Kulczycki score,[10] modified by omitting the chest X-ray evaluation. The highest possible score was therefore 75 points. Chest X-rays were assessed by Chrispin–Norman score.[11] Pulmonary function tests were performed in a whole-body plethysmograph (E. Jaeger, Würzburg, Germany). A sweat test was performed by quantitative pilocarpine iontophoresis.

Table 2. Comparison of clinical and laboratory data before antibiotic treatment (AB) and control phase (Co) at day 1

| | AB | Co | *p*-value |
|---|---|---|---|
| S–K score | 47.5 | 49.5 | n.s. |
| $FEV_1$ (% pred.) | 37 | 38 | n.s. |
| CRP (mg/ml) | 12.2 | 10.0 | n.s. |
| Bacteria/ml | $10^5$–$10^6$ | $10^6$–$10^7$ | <0.02 |
| MPO (μg/ml) | 238 | 315 | n.s. |
| Neutrophils/nl | 6.4 | 6.8 | n.s. |
| ECP (μg/ml) | 12.5 | 13.8 | n.s. |
| Eosinophils/nl | 0.00 | 0.15 | n.s. |

Numbers represent median values, n.s. = not significant.

Serum was prepared according to guidelines published recently,[11] and was stored at −20°C until analysed. First morning sputum samples were diluted 1:1 with phosphate-buffered saline (PBS). After mixing, 1 mg of DNase (Sigma, Deisenhofen, Germany) and 100 ml acetylcysteine was added. After vortexing, incubation was performed at 37°C for 30 to 60 min until complete liquifaction. Subsequently, samples were centrifuged for 10 min at 1 000 × **g**. The supernatants were stored at −80°C until analysis.

Eosinophil cationic proteins (ECP) and myeloperoxidase (MPO) were determined from serum and sputum supernatants by double antibody radioimmunoassay (Kabi Pharmacia, Uppsala, Sweden).[12–16] Sputum supernatants were diluted 1:100 before analysis.

Leucocyte counts were determined from peripheral EDTA-blood using a Coulter counter (Coulter Electronics, Krefeld, Germany). Eosinophil and neutrophil counts were calculated by differential blood cell count. C-reactive protein was analysed by nephelometry (Behringwerke, Marburg, Germany) using routine methods. Total and specific IgE was determined by solid phase immunoassay, using the 'Pharmacia CAP System' (Kabi Pharmacia, Uppsala, Sweden).[17]

*Statistical analysis:* For statistical analysis, the Wilcoxon matched-pairs signed rank and the Spearman correlation tests were used. A value of $p < 0.05$ was considered significant. Original data are given as medians and quartiles.

### Results

*Clinical parameters:* Modified Shwachman–Kulczycki scores improved significantly during antibiotic treatment from 47.5 on day 1 to 54.5 on day 12 ($p < 0.008$, Fig. 1A). In the control phase there were no statistically significant differences: 49.5 on day 1 to 50.5 on day 12. Comparison of antibiotic treatment and control phases revealed a statistically significant difference on day 12 ($p < 0.05$, Fig. 1A).

CRP decreased significantly during antibiotic treatment from 12.2 mg/l (4.9 – 23.8 mg/l) on day 1 to 6.2 mg/l (4.0 – 7.4 mg/l) on day 12 ($p < 0.05$, Fig. 1B). In the control phase no significant changes could be noted: 10.0 mg/l (6.2 – 13.0 mg/l) on day 1 to 11.4 mg/l (5.0 – 17.3 mg/l) on day 12.

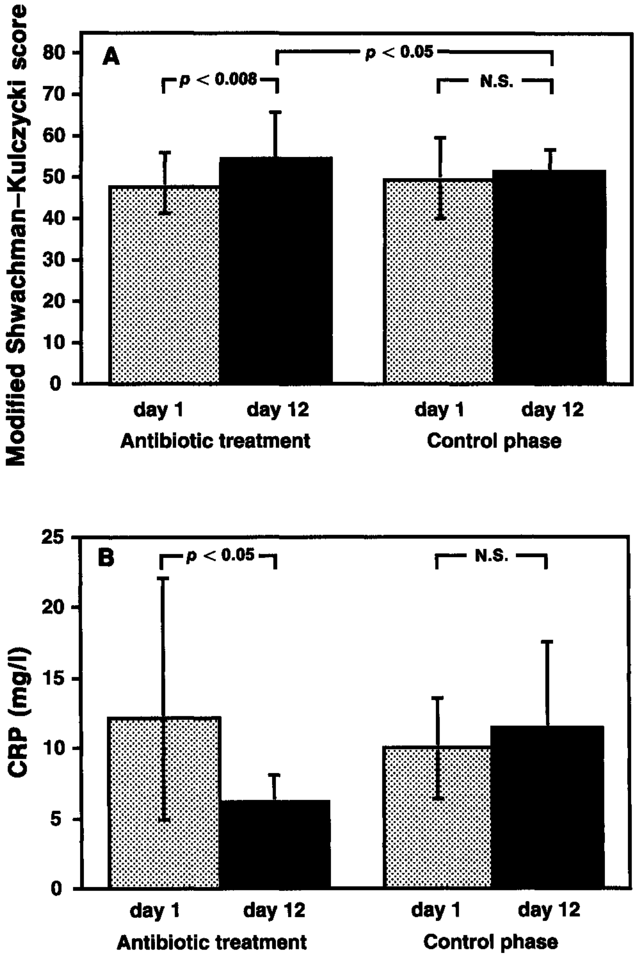

FIG. 1. Modified Shwachman-Kulczycki score (A) and C-reactive protein (B) on day 1 and day 12 during antibiotic treatment and control phase. Columns indicate median, bars indicate 25 and 75 percentiles.

Lung function (assessed by forced expiratory volume in 1 s ($FEV_1$) showed a tendency to increase during antibiotic treatment (from 37% of the predicted value on day 1 to 39% on day 12), while in the control phase there was a tendency to decrease (from 38% of the predicted value on day 1 to 32% on day 12) (Table 3). These results failed to reach significance. However, comparison of the antibiotic treatment and the control phase revealed a statistically significant difference for $FEV_1$ on day 12 ($p < 0.03$).

*Serum and peripheral blood data:* Serum MPO significantly decreased during antibiotic treatment from 238 µg/l on day 1 to 228 µg/l on day 5 ($p < 0.04$) and 175 µg/l on day 12 ($p < 0.006$, Table 3, Fig. 2A). In the control phase, no significant changes in serum MPO could be noted: 315 µg/l on day 1, 246 µg/l on day 5, and 262 µg/l on day 12 (Table 3, Fig. 2A). Comparison of the antibiotic treatment and the control phase revealed a statistically significant difference on day 12 ($p < 0.006$).

Peripheral blood neutrophil granulocyte counts decreased during antibiotic treatment from 6.4 cells/nl on day 1 to 6.1 cells/nl on day 5 ($p < 0.05$) and 4.7 cells/nl on day 12 ($p < 0.04$, Table 3, Fig. 2B). In the control phase no significant changes in neutrophil counts were seen: 6.8 cells/nl on day 1, 5.8 cells/nl on day 5, and 8.6 cells/nl on day 12 (Table 3, Fig. 2B).

Serum ECP concentration showed a tendency to decrease during antibiotic treatment from median 12.5 µg/l on day 1 to 9.5 µg/l on day 5 and 8.9 µg/l on day 12, but this failed to reach significance (Table 3, Fig. 3A). In the control phase, no changes in serum ECP could be noted: 13.8 µg/l on day 1, 12.2 µg/l on day 5, and 13.6 µg/l on day 12 (Table 3, Fig. 3A). Peripheral blood eosinophil counts increased during antibiotic treatment ($p < 0.03$) but not during the control phase (Table 3, Fig. 3B).

*Sputum data:* Microbial growth of *Pseudomonas aeruginosa* in sputum did not show a significant decrease both during the antibiotic treatment and the control phase (Table 3). In general, sputum concentrations of MPO and ECP were 500- to 1 000-fold higher than in serum (Table 4). There was no statistically significant correlation between serum and sputum MPO or between serum and sputum ECP concentration. Neither MPO nor ECP showed a significant variation over time during antibiotic treatment or the control phase.

Table 3. Clinical data as well as laboratory data in serum during antibiotic treatment (AB) and control phase (Co)

| | $FEV_1$ (%) | | Bacteria/ml | | S-K score | | CRP (mg/l) | | | Neutrophils/nl | | | MPO (mg/l) | | | Eosinophils/nl | | | ECP (mg/l) | | |
|---|---|---|---|---|---|---|---|---|---|---|---|---|---|---|---|---|---|---|---|---|---|
| | d 1 | d 12 | d 1 | d 12 | d 1 | d 12 | d 1 | d 5 | d 12 | d 1 | d 5 | d 12 | d 1 | d 5 | d 12 | d 1 | d 5 | d 12 | d 1 | d 5 | d 12 |
| 01 AB | 56 | 65 | $10^5 - 10^6$ | $10^5 - 10^6$ | 43 | 49 | 7.7 | 9.3 | 3.2 | 6.1 | 3.7 | 4.1 | 211 | 190 | 118 | 0.10 | 0.33 | 0.07 | 10.2 | 8.9 | 8.1 |
| Co | 45 | 45 | $10^6 - 10^7$ | $10^5 - 10^6$ | 36 | 50 | 10.9 | 15.2 | 25.0 | 10.2 | 6.9 | 6.7 | 404 | 415 | 320 | 0.13 | 0.20 | 0.00 | 13.3 | 20.3 | 13.1 |
| 02 AB | n.d. | n.d. | $10^6 - 10^7$ | $10^4$ | 25 | 39 | 33.0 | 16.7 | 9.5 | 5.4 | 6.1 | 6.7 | 288 | 268 | 208 | 0.00 | 0.12 | 0.30 | 23.2 | 10.4 | 22.9 |
| Co | n.d. | n.d. | $10^6 - 10^7$ | $10^6 - 10^7$ | 37 | 25 | 12.2 | 10.4 | 10.1 | 7.2 | 4.9 | 5.4 | 299 | 304 | 270 | 0.00 | 0.08 | 0.00 | 15.0 | 20.1 | 14.1 |
| 03 AB | 37 | 39 | $10^6 - 10^7$ | $10^6$ | 55 | 63 | 21.0 | 14.5 | 6.1 | 8.4 | 6.4 | 4.6 | 311 | 270 | 169 | 0.20 | 0.33 | 0.37 | 28.5 | 21.9 | 16.0 |
| Co | 38 | 34 | $10^6$ | $10^6 - 10^7$ | 50 | 55 | 15.4 | 9.2 | 18.0 | 3.8 | 3.8 | 4.0 | 429 | 246 | 348 | 0.12 | 0.30 | 0.13 | 12.2 | 7.6 | 15.3 |
| 04 AB | 63 | 73 | $10^6 - 10^7$ | $10^3$ | 46 | 55 | 4.7 | 4.6 | 6.8 | 4.8 | 3.2 | 4.0 | 152 | 164 | 146 | 0.62 | 1.26 | 0.83 | 36.4 | 45.9 | 48.9 |
| Co | 73 | 73 | $10^7 - 10^8$ | $10^4 - 10^5$ | 50 | 52 | 7.4 | 7.7 | 14.7 | 3.2 | 3.8 | 4.3 | 181 | 192 | 253 | 0.46 | 0.15 | 0.65 | 31.8 | 40.4 | 32.1 |
| 05 AB | 20 | 22 | $10^6$ | $10^6$ | 35 | 37 | 5.0 | 27.2 | 6.6 | 6.4 | 6.5 | 5.9 | 233 | 251 | 189 | 0.00 | 0.20 | 0.29 | 14.7 | 16.3 | 9.7 |
| Co | 17 | 20 | $10^6 - 10^7$ | $10^7 - 10^8$ | 39 | 44 | 8.4 | 2.5 | 5.9 | 6.7 | 6.1 | 9.2 | 331 | 240 | 271 | 0.62 | 0.09 | 0.25 | 21.2 | 10.5 | 11.4 |
| 06 AB | 27 | 30 | $10^5 - 10^6$ | $10^6 - 10^7$ | 55 | 65 | 14.1 | 12.0 | 2.4 | 9.3 | 7.0 | 5.3 | 283 | 296 | 188 | 0.27 | 0.10 | 0.39 | 8.9 | 9.2 | 5.9 |
| Co | 33 | 22 | $10^6$ | $10^5 - 10^6$ | 63 | 55 | 10.9 | 5.9 | 11.2 | 5.2 | 5.4 | 9.7 | 220 | 176 | 242 | 0.28 | 0.65 | 0.40 | 9.3 | 4.0 | 8.3 |
| 07 AB | n.d. | n.d. | $10^4$ | $10^6$ | 65 | 65 | 2.4 | 3.1 | 9.2 | 7.8 | 7.0 | 4.7 | 137 | 122 | 134 | 0.00 | 0.22 | 0.33 | 3.5 | 5.9 | 5.7 |
| Co | n.d. | n.d. | $10^6$ | $10^6$ | 62 | 62 | 2.4 | 2.7 | 2.4 | 9.0 | 7.4 | 8.6 | 160 | 172 | 156 | 0.00 | 0.00 | 0.12 | 3.8 | 6.8 | 3.6 |
| 08 AB | n.d. | n.d. | $10^5 - 10^6$ | $10^3 - 10^4$ | 49 | 54 | 32.0 | 16.3 | 5.2 | 13.2 | 6.0 | 6.6 | 168 | 146 | 110 | 0.00 | 0.10 | 0.28 | 8.4 | 6.3 | 6.4 |
| Co | n.d. | n.d. | $10^7 - 10^8$ | $10^5 - 10^6$ | 49 | 49 | 30.1 | 11.2 | 17.0 | 6.8 | 6.4 | 7.6 | 346 | 324 | 216 | 0.10 | 0.33 | 0.00 | 12.7 | 13.9 | 8.7 |
| 09 AB | 42 | 39 | $10^4 - 10^5$ | $10^6 - 10^7$ | 61 | 65 | 10.3 | 2.8 | 4.3 | 4.6 | 3.9 | 4.4 | 242 | 205 | 181 | 0.00 | 0.07 | 0.40 | 8.3 | 9.2 | 7.3 |
| Co | 38 | 32 | $10^8$ | $10^7$ | 59 | 59 | 2.3 | 2.3 | 3.5 | 2.9 | 2.7 | 2.1 | 187 | 164 | 210 | 0.17 | 0.30 | 0.12 | 19.2 | 10.3 | 16.3 |
| 10 AB | 20 | 23 | $10^5 - 10^6$ | $10^5 - 10^6$ | 46 | 52 | 14.5 | 6.9 | 6.3 | 10.5 | 13.1 | 7.5 | 681 | 405 | 320 | 0.00 | 0.00 | 0.10 | 26.4 | 17.5 | 16.9 |
| Co | 20 | 20 | $10^7 - 10^8$ | $10^5 - 10^6$ | 47 | 45 | 9.1 | 12.6 | 5.5 | 9.4 | 8.1 | 10.5 | 350 | 363 | 416 | 0.44 | 0.93 | 0.75 | 14.2 | 21.1 | 18.0 |
| median | 37 | 39 | $10^5 - 10^6$ | $10^5$ | 47.5 | 54.5 | 12.2 | 10.5 | 6.2 | 6.4 | 6.1 | 4.7 | 238 | 228 | 175 | 0.00 | 0.16 | 0.32 | 12.5 | 9.5 | 8.9 |
| | 38 | 32 | $10^6$ | $10^5 - 10^6$ | 49.5 | 50.5 | 10.0 | 8.5 | 11.4 | 6.8 | 5.8 | 8.6 | 315 | 246 | 262 | 0.14 | 0.25 | 0.13 | 13.8 | 12.2 | 13.6 |

n.d. = not determined, S-K score = Shwachman-Kulczycki score

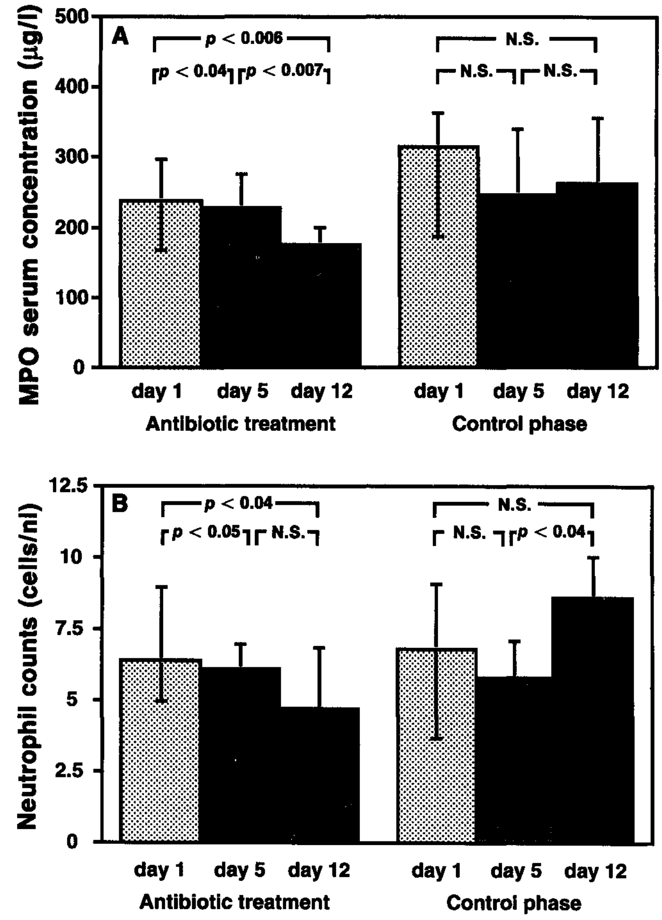

FIG. 2. Serum MPO concentration (A) and peripheral blood neutrophil granulocyte counts (B) during antibiotic treatment and control phase. Columns indicate median, bars indicate 25 and 75 percentiles.

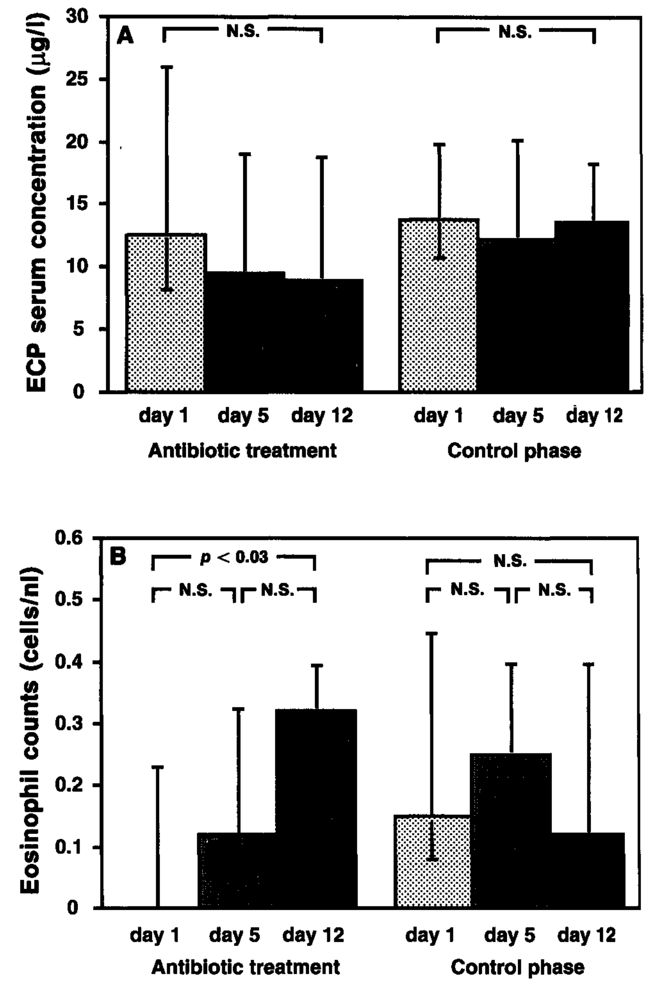

FIG. 3. Serum ECP concentration (A) and peripheral blood eosinophil counts (B) during antibiotic treatment and control phase. Columns indicate median, bars indicate 25 and 75 percentiles.

### Discussion

Originally, a placebo-controlled design was intended and the present study was started in this manner. However, after two patients suffered from pulmonary exacerbations during a placebo 'treatment', the study was continued as an intra-individual cross-over design (each patient serving as its own control). Conditions during the antibiotic treatment were completely comparable (e.g., in terms of physiotherapy, inhalation therapy, dietetic intervention) with those during the control phase, with the exception of the intravenous treatment.

The reason for choosing a control-phase in addition to antibiotic treatment was the possible bias of the admission to hospital, which may improve a patient's well-being by intensified basic therapy. In order to minimize observer bias, the investigator of the study was not the physician responsible for the care of the patients on the ward.

In order to study the monitoring character of the parameters investigated, blood was sampled

Table 4. MPO and ECP concentrations in sputum during antibiotic treatment (AB) and control phase (Co)

| Sputum | | day 1 | day 5 | day 12 |
|---|---|---|---|---|
| MPO (mg/l) | AB | 105.8 (44.4 - 176.1) | 69.3 (31.0 - 148.6) | 103.0 (47.8 - 150.2) |
| | Co | 205.4 (106.2 - 234.8) | 162.6 (97.0 - 213.1) | 163.9 (103.3 - 224.9) |
| ECP (mg/l) | AB | 4.3 (2.0 - 5.8) | 1.6 (1.0 - 7.9) | 2.6 (2.0 - 6.5) |
| | Co | 4.3 (2.9 - 7.3) | 3.9 (1.9 - 5.1) | 4.2 (1.6 - 6.9) |

Numbers represent median values; in brackets: 25 and 75 percentile. Differences between day 1 of antibiotic treatment and day 1 of control phase were not statistically significant for any of the investigated parameters.

on 7 days during the antibiotic treatment and control phases. All data points of each individual were analysed; however, three time-points (day 1, 5 and 12) are given in the figures and tables to avoid unclear presentation and because complete data did not add any information to those presented.

Lung function parameters, such as $FEV_1$, did not differ significantly on day 1 either before starting antibiotic treatment (median 37% of predicted) or control phase (median 38% of predicted). The same was true for modified Shwachman–Kulczycki scores: median 47.5 points for day 1 of antibiotic treatment and 49.5 points for day 1 of the control phase. Therefore, starting points for both phases were comparable (Table 2).

$FEV_1$ values showed an improvement at the end of antibiotic treatment compared with the control phase. This effect of antibiotic treatment has been observed by other authors.[6,18,19] The improvement of lung function was paralleled by other parameters such as the Shwachman–Kulczycki score, serum MPO concentration and CRP. It seems that this positive effect is even seen at a time-point where patients did not suffer from clinical exacerbations.

CRP reflects the pulmonary inflammatory state in chronically infected CF patients, is increased during pulmonary exacerbations, and decreases during antibiotic treatment,[6] but it is not a reliable indicator of intermittent bacterial colonization in early lung disease.[20] Very high concentrations of CRP are seen days and weeks before death.[21] However, in bronchoalveolar lavage (BAL), the degree of airway obstruction, neutrophil elastase activity, myeloperoxidase activity, or total neutrophils did not correlate with the density of *P. aeruginosa* (CFU/ml) or total pathogen burden in BAL fluid.[22]

MPO, as a neutrophil inflammatory mediator, is not only increased in chronically infected cystic fibrosis patients, but also in other diseases. It was found increased in BAL of idiopathic pulmonary fibrosis,[23] transiently increased in BAL during recovery from airway hyperresponsiveness[24] and increased in BAL from some patients with bronchial asthma.[25] Furthermore, the release of MPO from neutrophils of patients with asthma was somewhat higher than in controls.[26] Serum MPO concentration was even suggested to be a good indicator of exposure to noxious agents causing respiratory disorders, such as in 'sick building syndrome'.[27] From this study, there is evidence for a role of MPO in chronic lung inflammation in CF, as shown by elevated blood levels in some patients, as well as the impact of antibiotic treatment on serum MPO concentration.

Concerning the role of the eosinophil and its granule proteins in CF, there is little data in the literature: Koller *et al.* found increased serum levels of ECP in most of their 42 CF patients.[7] In contrast, only three of our ten CF patients had an increased serum ECP concentration (>20 μg/l), although CF in our patients was more severe than in the Austrian group. The four atopic patients did not show a different pattern from the non-atopic patients.

Conflicting results continue, e.g., Azzawi *et al.* observed an increased number of EG2 positive cells in post-mortem lungs of CF patients, but normal numbers of eosinophils.[8] This discrepancy may be interpreted as an increased propensity of the eosinophils to release their granule proteins as suggested by Koller.[7] Serum ECP levels showed a tendency to decrease under antibiotic treatment, but this failed to reach significance in either the Austrian study (elevated levels of ECP in 90% of their patients), or in our investigation (30% of patients had an increased ECP concentration).

Peripheral blood eosinophils increased during antibiotic treatment in our study. Because of the low absolute numbers of eosinophils, our observation is probably not of clinical relevance, despite the statistical significance. Further studies are needed to elucidate the role of the eosinophil for airway inflammation in CF.

In the local compartment, the sputum, MPO as well as ECP concentrations, were 500- to 1 000-fold higher than in serum. This reflects their role in local inflammatory lung injury. However, the failure to reduce sputum concentrations of these highly toxic proteins by intravenous antibiotic therapy makes additional forms of treatment necessary. The Austrian group found a significant correlation between serum and sputum MPO and ECP concentration.[7] Our data does not confirm this. The inherent problem lies in the non-homogenous composition of sputum, depending on local factors of the lung region from which it was sampled. In our view, sputum measurements do not seem to be a reliable parameter for monitoring inflammatory changes in CF.

From our data we conclude that: (1) measurements of MPO, neutrophils and CRP in peripheral blood correlate with clinical parameters such as the modified Shwachman–Kulczycki score; (2) neutrophils and MPO seem to reflect inflammatory changes induced by antibiotic treatment; (3) eosinophils may play a role in CF by an enhanced 'releasability'; and (4) sputum measurements of mediators of inflammation cannot be recommended.

### References

1. Bruce MC, Poncz L, Klinger JD, Stern RC, Tomashefski JF jr, Dearborn DG. Biochemical and pathologic evidence for proteolytic destruction of lung connective tissue in cystic fibrosis. *Am Rev Respir Dis* 1985; **132:** 529–535.
2. Suter S, Schaad UB, Roux L, Nydegger UE, Waldvogel FA. Granulocyte neutral proteases and *Pseudomonas elastase* as possible causes of airway damage in patients with cystic fibrosis. *J Infect Dis* 1984; **4:** 523–531.
3. Tetley TD. Proteinase imbalance: its role in lung disease. *Thorax* 1993; **48:** 560–565.
4. Elborn JS, Cordon SM, Shale DJ. Host inflammatory responses to first isolation of *Pseudomonas aeruginosa* from sputum in cystic fibrosis. *Pediatr Pulmonol* 1993; **15:** 287–291.
5. Cantin A, Woods DE. Protection by antibiotics against myeloperoxidase-dependent cytotoxicity to lung epithelial cells *in vitro*. *J Clin Invest* 1993; **91:** 38–45.
6. Rayner RJ, Wiseman MS, Cordon SM, Norman D, Hiller EJ, Shale DJ. Inflammatory markers in cystic fibrosis. *Resp Med* 1991; **85:** 139–145.
7. Koller DY, Götz M, Eichler I, Urbanek R. Eosinophilic activation in cystic fibrosis. *Thorax* 1994; **49:** 496–499.
8. Azzawi M, Johnston PW, Majumdar S, Kay AB, Jeffrey PK. T lymphocytes and activated eosinophils in airway mucosa in fatal asthma and cystic fibrosis. *Am Rev Respir Dis* 1992; **145:** 1477–1482.
9. Hoiby N, Koch C. *Pseudomonas aeruginosa* infection in cystic fibrosis and its management. *Thorax* 1990; **45:** 881–884.
10. Shwachman H, Kulczycki LL. Long-term study of one hundred and five patients with cystic fibrosis. *Am J Dis Child* 1958; **96:** 6–15.
11. Chrispin AR, Norman AP. The systematic evaluation of the chest radiograph in cystic fibrosis. *Pediat Radiol* 1974; **2:** 101–106.
12. Peterson CGB, Enander I, Nystrand J, Anderson AS, Nilson L, Venge P. Radioimmunoassay of human eosinophil cationic protein (ECP) by an improved method. Establishment of normal values in serum and turnover *in vivo*. *Clin Exp Allergy* 1991; **21:** 561–567.
13. Venge P, Roxin LE, Olsson I. Radioimmunoassay of human eosinophil cationic protein. *Br J Haematol* 1977; **37:** 331–335.
14. Enander I, Andersson AS, Peterson C, Venge P. A new radio-immunoassay to determine human neutrophilic myeloperoxidase (MPO). *Schweiz Med Wschr* 1991; **121**(Suppl 40): 38.
15. Venge P, Stromberg A, Brocanier JH, Roxin LE, Olsson I. Neutrophil and eosinophil granulocytes in bacterial infection: sequential studies of cellular and serum levels of granule proteins. *Br J Haematol* 1978; **38:** 475–483.
16. Olofsson T, Olsson I, Venge P, Elgefors B. Serum myeloperoxidase and lactoferrin in neutropenia. *Scand J Haematol* 1977; **18:** 73–88.
17. Axén R, Drevin H, Kober A, Yman L. A new laboratory diagnostic system applied to allergy testing. *New Engl Reg Allergy Proc* 1988; **9:** 503.
18. Regelmann WE, Elliott GR, Warwick WJ, Clawson CC. Reduction of sputum *Pseudomonas aerugonisa* density by antibiotics improves lung function in cystic fibrosis more than bronchodilators and chest physiotherapy alone. *Am Rev Respir Dis* 1990; **141:** 914–921.
19. Pedersen SS, Pressler T, Pedersen M, Hoiby N, Friis-Möller A, Koch C. Immediate and prolonged clinical efficacy of ceftazidime versus ceftazidime plus tobramycin in chronic *Pseudomonas aeruginosa* infection in cystic fibrosis. *Scand J Infect Dis* 1986; **18:** 133–137.
20. Watkin SL, Elborn JS, Cordon SM, Hiller EJ, Shale DJ. C-reactive protein is not a useful indicator of intermittent bacterial colonization in early lung disease of patients with cystic fibrosis. *Pediatr Pulmonol* 1994; **17:** 6–10.
21. Elborn JS, Cordon SM, Parker D, Delamere FM, Shale DJ. The host inflammatory response prior to death in patients with cystic fibrosis and chronic *Pseudomonas aeruginosa* infection. *Resp Med* 1993; **87:** 603–607.
22. Meyer KC, Zimmerman J. Neutrophil mediators, *Pseudomonas*, and pulmonary dysfunction in cystic fibrosis. *J Lab Clin Med* 1993; **121:** 654–661.
23. Hällgren R, Bjermer L, Lundgren R, Venge P. The eosinophil component of the alveolitis in idiopathic pulmonary fibrosis. *Am Rev Respir Dis* 1989; **139:** 373–377.
24. Gundel RH, Wegner CD, Letts LG. The onset and recovery from airway responsiveness: relationship with inflammatory infiltrates and release of cytotoxic granule proteins. *Clin Exp Allergy* 1992; **22:** 303–308.
25. Bousquet J, Chanez P, Lacoste JY, *et al.* Indirect evidence of bronchial inflammation assessed by titration of inflammatory mediators in Bal fluid of patients with asthma. *J Allergy Clin Immunol* 1991; **88:** 649–660.
26. Carlson M, Håkansson L, Peterson C, Stålenheim G, Venge P. Secretion of granule proteins from eosinophils and neutrophils is increased in asthma. *J Allergy Clin Immunol* 1991; **87:** 27–33.
27. Metso T, Björksten F, Kilpiö K, Kiviranta K, Haahtela T. Serum myeloperoxidase and sick building syndrome. *Lancet* 1993; **342:** 113–114.

**Received 9 March 1995; accepted in revised form 1 May 1995**
